# Transcriptome Profile of *Trichoderma harzianum* IOC-3844 Induced by Sugarcane Bagasse

**DOI:** 10.1371/journal.pone.0088689

**Published:** 2014-02-18

**Authors:** Maria Augusta Crivelente Horta, Renato Vicentini, Priscila da Silva Delabona, Prianda Laborda, Aline Crucello, Sindélia Freitas, Reginaldo Massanobu Kuroshu, Igor Polikarpov, José Geraldo da Cruz Pradella, Anete Pereira Souza

**Affiliations:** 1 Center for Molecular Biology and Genetic Engineering (CBMEG), University of Campinas (UNICAMP), Campinas, SP, Brazil; 2 Brazilian Bioethanol Science and Technology Laboratory (CTBE), Brazilian Center of Research in Energy and Materials (CNPEM), Campinas, SP, Brazil; 3 Physics Institute of São Carlos, University of São Paulo (USP), São Carlos, SP, Brazil; 4 Institute of Science and Technology, Federal University of São Paulo (UNIFESP), São José dos Campos, SP, Brazil; 5 Department of Plant Biology, Biology Institute, University of Campinas (UNICAMP), Campinas, SP, Brazil; University of South Florida College of Medicine, United States of America

## Abstract

Profiling the transcriptome that underlies biomass degradation by the fungus *Trichoderma harzianum* allows the identification of gene sequences with potential application in enzymatic hydrolysis processing. In the present study, the transcriptome of *T. harzianum* IOC-3844 was analyzed using RNA-seq technology. The sequencing generated 14.7 Gbp for downstream analyses. *De novo* assembly resulted in 32,396 contigs, which were submitted for identification and classified according to their identities. This analysis allowed us to define a principal set of *T. harzianum* genes that are involved in the degradation of cellulose and hemicellulose and the accessory genes that are involved in the depolymerization of biomass. An additional analysis of expression levels identified a set of carbohydrate-active enzymes that are upregulated under different conditions. The present study provides valuable information for future studies on biomass degradation and contributes to a better understanding of the role of the genes that are involved in this process.

## Introduction

The fungus *Trichoderma harzianum* is a well-known biocontrol agent [1],[2]. Most previously published genetic studies concerning this organism have explored its molecular mechanisms of biocontrol. This biocontrol ability enables the fungus to identify and degrade cell walls, and the mechanisms that underlie these processes were explored in the present study.

Several studies have suggested that *T. harzianum* may be utilized for the production of hydrolytic enzymes from a cellulolytic complex [3],[4],[5],[6], due to its ability to produce high levels of both β-glucosidase and endoglucanases [7]. These studies have demonstrated that this fungus is a potential source of hydrolytic enzymes and may aid in understanding the transcriptional regulation of biomass degradation by filamentous fungi. The utilization of sugarcane bagasse as a biomass for the production of second-generation ethanol requires its degradation into mono-oligosaccharides and small oligosaccharides that may be metabolized by ethanol-producing yeast. The major bottleneck for this process is the enzymatic hydrolysis of sugarcane bagasse [8]. The hydrolytic effectiveness of an enzymatic mixture is highly dependent on the feedstock and any pretreatment it has received [9]. A strategic issue to be considered during the development of enzymatic mixtures optimized for second-generation ethanol production is the cultivation of microorganisms utilizing the lignocellulosic material that will be hydrolyzed. This cultivation method may select for enzymes that are optimal for the hydrolysis of a specific feedstock [9],[10]. One of the primary mechanisms of the adaptive processes of cells in a complex medium is the alteration of transcription levels, which can lead to the production of specialized proteins, differences in membrane composition and other changes in cellular machinery [11].

A large variety of enzymes with different specificities are required to degrade the components of lignocellulose [10],[12],[13],[14]. However, many other proteins may also contribute to lignocellulose degradation in ways that are not yet clearly understood, such as the glycoside hydrolase family 61 proteins, the expansins and the swollenins [10],[14],[15]. Three types of enzymes are required to hydrolyze cellulose into glucose monomers: exo-1,4-β-glucanases, such as EC 3.2.1.91 and EC 3.2.1.176 (cellobiohydrolase); endo-1,4-β-glucanases, such as EC 3.2.1.4; and β-glucosidases, such as EC 3.2.1.21 (cellobiases) [10],[16]. Cellobiohydrolases attack the reducing or nonreducing ends of the cellulose chains, whereas endo-glucanases cleave these chains in the middle and reduce the degree of polymerization [10],[17]. The composition of hemicellulose is more variable than that of cellulose; therefore, more enzymes are required for its effective hydrolysis. The enzymes that degrade hemicellulose can be divided into depolymerizing enzymes, which cleave the backbone of the molecule, and enzymes that remove the substituent of the molecule, which may sterically hinder the depolymerizing enzymes. The core enzymes for the degradation of xylan to monomers are the endo-xylanases, which cleave the xylan backbone into shorter oligosaccharides, and β-xylosidase, which cleaves short xylo-oligosaccharides into xylose. Similarly, the core enzymes for the degradation of mannan are endo-mannanase and β-mannosidase. However, xylans and mannans generally contain a number of different substituents linked to their main backbones, including arabinose, acetyl groups, galactose and glucose. A host of ancillary enzymes are required to remove these substituents and allow the core enzymes to degrade the xylan and mannan backbones. These ancillary enzymes include the α-L-arabinofuranosidases, α-glucuronidase, ferulic acid esterase, α-galactosidase, feruloyl esterase, acetyl xylanesterase and acetyl mannan esterase. The ferulic acid esterases specifically cleave the linkages between hemicellulose and lignin. The α-L-arabinofuranosidases also possess different specificities; some cleave 1,2 linkages or 1,3 linkages, whereas others cleave doubly substituted arabinose residues from arabinoxylan [10],[18].

Fungi from the genera *Trichoderma*, *Penicillium*, *Aspergillus* and *Humicola grisea var. thermoidea* degrade lignocellulose components, including sugarcane bagasse [8]. These fungi can degrade cellulose, hemicellulose and lignin in decaying plants using a complex set of excreted hydrolytic and oxidative enzymes, including glycosyl hydrolases from different families [10]. Although many studies have been conducted to characterize the action of the enzymes involved in lignocellulose degradation, little is known regarding the transcription and genomic regulation of the genes that encode these enzymes. *Trichoderma reesei* is the major industrial source of the cellulases and hemicellulases that are utilized in the depolymerization of biomass to simple sugars, which are then further converted into chemical intermediates and biofuels. Unexpectedly, despite the industrial utility and effectiveness of the carbohydrate-active enzymes of *T. reesei*, the genome of this species encodes fewer cellulases and hemicellulases than that of any other sequenced fungus that can hydrolyze plant cell wall polysaccharides [19],[20]. Thus, a better understanding of the genetic mechanisms of this fungus is necessary to explore its extraordinary biotechnological potential. The present study analyzes the transcriptome of *T. harzianum* IOC-3844 grown in a sugarcane bagasse-based culture medium and the induction of hydrolytic activity in this medium, with particular emphasis on the potential contributions of the fungus to fuel biotechnology and other industrial applications. This organism is available in public collections, and studies addressing the mechanisms of regulating and gene expression in this fungus are important to make its use in biotechnological processes viable. This work seeks to contribute to the understanding of the reactions involved in biomass degradation at the enzymatic level and will serve as the basis for other studies exploring the biotechnological potential presented by *T. harzianum*. The primary goal of these analyses was to identify, characterize and catalog the transcripts expressed by *T. harzianum* that are involved in the degradation of complex substrates, thereby revealing the complexity of the hydrolytic pathways involved in biomass degradation.

## Materials and Methods

Regarding the Ethics Statement, we confirm that no specific permits were required for the present studies. Additionally, we confirm that the field studies did not involve endangered or protected species.

### Strain and Culture Media

The *T. harzianum* IOC-3844 strain used in this study was provided by Professor Dr. Nei Pereira Jr. (Federal University of Rio de Janeiro, Rio de Janeiro, Brazil). The species was confirmed by comparing its ITS1 and ITS2 sequences with those of standard strains of *T. harzianum*. (available at Institute Oswaldo Cruz, Rio de Janeiro, RJ, Brazil and Centro de Pesquisas Químicas e Biológicas na Agricultura (CPQBA) - CBMAI, UNICAMP, Campinas, SP, Brazil). The stock cultures were stored at 4°C on potato dextrose agar (PDA) slants. The fungi were grown on PDA plates (90×15 mm) at 29°C for 8 days.

The composition of the basal medium was adapted from Mandels and Weber (1969) [21] and included (g L^−1^) KH_2_PO_4_ (2.0), NH_4_SO_4_ (1.4), MgSO_4_·7H_2_O (0.3), CaCl_2_·2H_2_O (0.3), CoCl_2_ (0.002), MnSO_4_·H_2_O (0.0016), ZnSO_4_·H_2_O (0.0014), FeSO_4_·7H_2_O_4_ (0.005) and urea (0.3). The pH was adjusted to 5.2. Three different preculture media for mycelial production were prepared from the basal medium through the addition of 2 g L^−1^ glucose, 1 g L^−1^ peptone, 1 mL L^−1^ Tween 80 and 10 g L^−1^ of a carbon source. The carbon sources used in the three preculture media were lactose, crystalline cellulose and delignified sugarcane bagasse (DSB, from a local mill, Usina Vale do Rosário, Orlândia, SP, Brazil), which was prepared and characterized according to Rocha *et al.* (2012) [22]. The percentage composition of the DSB was 89.5±1.6 cellulose, 3.4±0.3 hemicellulose and 5.5±0.2 lignin [4]. The preculture media were sterilized at 121°C for 20 min.

The production medium was composed of the basal medium, 10 g L^−1^ DSB as a unique carbon source, 1 g L^−1^peptone and 1 mL L^−1^ Tween 80; the medium was then sterilized at 121°C for 20 min. All other chemicals were of at least analytical grade. The following libraries were classified based on the preculture media: the “DSB” library was generated from a preculture medium that contained DSB, the “CEL” library was generated from a preculture medium that contained crystalline cellulose, and the “LAC” library was generated from a preculture medium that contained lactose. This latter condition was designated as the control.

### Preculture and fermentation

Conidial suspensions were prepared through the addition of sterilized distilled water and Tween 80 to the PDA plates, which resulted in conidial suspensions of 9×10^5^ spores mL^−1^. After preparation, 4.0 mL of each conidial suspension was transferred to Erlenmeyer flasks containing 600 mL of each preculture medium, and the flasks were incubated for 72 h at 29°C on a rotary shaker at 200 rpm. A volume of 30 mL of each medium was transferred to individual Erlenmeyer flasks containing 270 mL of the production medium. The flasks were incubated at 29°C for 129 h on a rotary shaker at 200 rpm. Samples of the mycelia and the fermentation extracts were removed to determine the enzymatic activity and to conduct the transcriptome analyses.

### Analytical measurements

The filter paper activity (FPase) was determined as described by Ghose (1987) [23], with modifications to diminish the scale of the procedure by a factor of 10. All statistical comparisons were done using Student's t test (P<0.05).

### RNA extraction and transcriptome sequencing

The mycelial samples from the LAC, CEL and DSB conditions were extracted after 96 h of fermentation, stored at −70°C and used for RNA extraction. The fungal mRNA was isolated according to Jones *et al.* (1985) [24] with some modifications. Two grams of each mycelial sample was frozen using liquid nitrogen in a mortar and ground with a pestle into a fine powder. Next, NTES buffer (4.5 mL, 0.1 M NaCl, 0.01 M Tris-HCl at pH 7.5, 1 mM EDTA and 1% SDS) and phenol/chloroform/isoamyl alcohol [3 mL of a 1∶1∶1 mixture] were added, and the sample was ground until the mixture had thawed. After vortexing for 10 min, the solution was centrifuged at 8,000 rpm for 10 min at 4°C. To the aqueous phase, 3 mL of phenol/chloroform/isoamyl alcohol [1∶1∶1 mixture] was added, and the solution was centrifuged at 8,000 rpm for 10 min. The aqueous phase was then removed, and the nucleic acid was precipitated through the addition of a 0.1 volume aliquot of 2 M NaAc, pH 4.5, and two volumes of 100% ethanol. The precipitate was centrifuged at 8,000 rpm for 10 min, and the pellet was resuspended in 2.5 mL of sterile water. To remove the DNA, 2.5 mL of 4 M LiAc was added, and the solution was incubated for 48 h at −20°C. The precipitate was collected by centrifugation at 8,000 rpm for 10 min, then washed with 70% ethanol, resuspended in 50 µL of sterile water and stored at −70°C.

The RNA samples were quantitated using a fluorescence-based method, and their quality was determined using a 2100 Bioanalyzer (Agilent Technologies, Palo Alto, CA).

The libraries were constructed using 4 µg of each RNA sample and the TruSeq RNA sample preparation kit (Illumina Inc., San Diego, CA) according to the manufacturer's instructions. The expected target sizes were confirmed using a 2100 Bioanalyzer (Agilent Technologies, Palo Alto, CA), and the libraries were quantified using qPCR. The average insertion size was 260 bp. The clustering was conducted using 10 µM of each library and a TruSeq PE Cluster Kit on cBot (Illumina Inc., San Diego, CA). The sequencing was performed on the Illumina Genome AnalyzerIIx, which is a next-generation high-throughput sequencer (Illumina Inc., San Diego, CA), according to the manufacturer's specifications for paired-end reads of 72 bp in individual lanes.

### Downloading and processing the sequence data

After the sequencing was completed, the data were transferred to a local high-performance computing server at CBMEG (University of Campinas, Campinas, Brazil). The results were submitted to NCBI under accession number SRX189214, and the raw sequences (archives of paired and paired-end sequences) were submitted to the NCBI Sequence Read Archive (SRA) under accession numbers SRR579379, SRR631745 and SRR631746 for the DSB, CEL and LAC libraries, respectively. Initially, all sequences were trimmed. We utilized the CLC Genomics Workbench (v4.0; Finlandsgade, Dk) to perform the reads trimming, and parameters were set to: quality limit: 0,03; ambiguous limit: 2; minimum final number of nucleotides in reads: 65; phred scale: 15. *De novo* assembly was conducted using CLC Genomics Workbench (v4.0; Finlandsgade, Dk) with the following parameters: similarity = 0.98 and length fraction = 0.9. The resulting contigs were compared with the NCBI non-redundant protein database (NR) using BLAST to identify homologous sequences [25], with an *E*-value cutoff of ≤l e^−5^. The sequences were functionally annotated according to Gene Ontology terms [26], and the annotations were compared with the Kyoto Encyclopedia of Genes and Genomes [28] to establish biochemical pathway associations using Blast2Go, which is a universal web-based annotation application [27] The sequences were aligned against the Carbohydrate-Active Enzymes (CAZymes) database to identify glycosyl hydrolases [29],[30],[31],[32],[33], glycosyltransferases [34],[35], carbohydrate-binding modules [36] and carbohydrate esterases [37].The *T. harzianum* IOC-3844 genome was provided by Dr. Reginaldo M. Kuroshu (University of São Paulo, São Carlos, Brazil). Only the best alignments showing expectation values lower than 1×10^−5^ were considered for functional gene annotation. To compare the transcript sequences with the genome and CAZyme datasets, CLC Genomics Workbench was used. The archive of the assembly scaffolds for *T. harzianum* CBS 226.95, which is available on JGI (sequence data produced by the US Department of Energy Joint Genome Institute in collaboration with the user community) [38], was used to calculate the similarity between the data.

### Expression pattern

A paired Kal's *t*-test was conducted on the log_2_-transformed data to determine whether significant differences existed between the expression ratios found in each treatment and the control. Contigs were identified as being differentially expressed in upregulated groups when significance was detected with a false discovery rate lower than 1×10^−3^. Hierarchical clustering analysis and K-means clustering were performed on the CAZymes that were identified as being differentially expressed. Clustering was performed using Euclidean distance as the distance metric in three partitions according to the cluster features, on the transformed expression values.

### Real-Time PCR analysis

To validate the expression profiles of the assembled genes obtained through sequencing data analysis, quantitative real-time (RT-qPCR) was performed for selected genes. Genes associated with biomass degradation processes were selected and are shown in Table S1, together with the primers and annealing temperatures.

**Figure 1:**
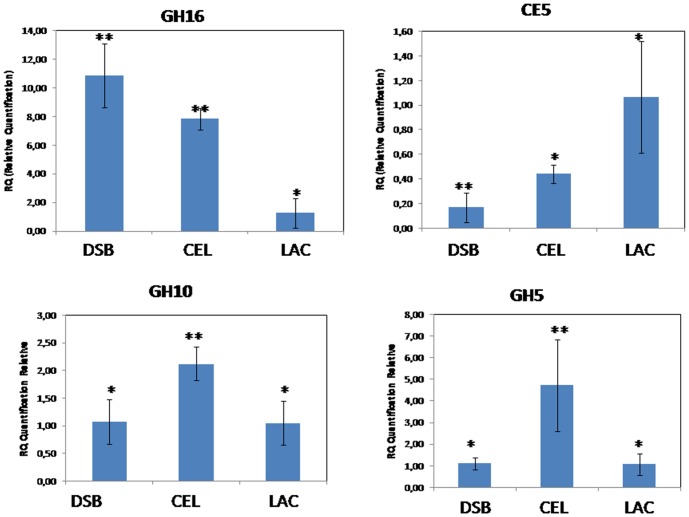
Expression profiles of selected glycosyl hydrolase genes determined by RT-qPCR. The squalene-epoxidase gene was used as endogenous control. The differences between groups were considered significant at P<0.05 (Student's t test) and are indicated by *.

Quantification of gene expression was performed by continuously monitoring SYBR Green fluorescence. The reactions were performed in triplicate in a total volume of 6.25 µl. Each reaction included 3.12 µl of SYBR Green Master Mix (Invitrogen, Carlsbad, CA), 1.0 µl of direct and reverse primers, 0.5 µL of cDNA and 1.6 µl of water. The reactions were assembled in 384-well plates. PCR amplification-based expression profiling of the selected genes was performed using a gene for squalene-epoxidase as endogenous control. Four genes were tested as endogenous control: genes for actin, beta-tubulin, glyceraldehyde 3-phosphate dehydrogenase, and squalene-epoxidase. The last one had the best performance in RT-qPCR analysis, remaining constant in all treatments. The enzyme squalene-epoxidase catalyses the conversion of squalene to 2,3-(S) oxidosqualene, which is an intermediate in the synthesis of the fungal cell membrane component ergosterol. RT-qPCR was conducted in an ABI PRISM 7500 HT (Applied Biosystems, Foster City, CA). Gene expression was calculated via the Delta-Delta cycle threshold method [47]. All statistical comparisons were done using Student's t test (P<0.05). The obtained RT-qPCR results were in agreement with the RNA expression analyses of the generated assemblies. The same expression profile was observed for the genes encoding GH16, GH10, CE5, and GH5. Figure 1 shows the expression of the selected genes.

## Results

### Enzymatic Activity Profile

**Figure 2:**
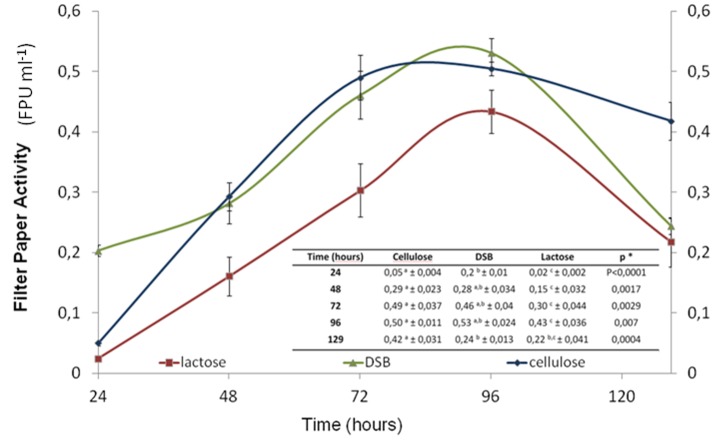
Filter paper activity enzymatic profiles (FPU mL^−1^ h^−1^). *T. harzianum* fermentation on a complex substrate (DSB) from extracts grown on preculture media using DSB (▵), cellulose (◊) or lactose (□) as the carbon source. The differences were considered significant at P<0.05 (Student's t-test) and are indicated by (a) for cellulose, (b) for DSB and (c) for lactose samples.

The FPase was evaluated to determine the enzymatic activity profile of the cellulases during 129 h of fermentation (Figure 2) using DSB as a carbon source. RNA was isolated from the mycelia at 96 h of cultivation; this time point was associated with a significant production of FPase (0.53 FPU mL^−1^) (Figure 2). This 96 h cultivation period included a 48 h adaptation phase. Previous studies have indicated that the enzymatic activity of this fungus, as measured by cellulase production, is lower when grown on soluble carbon sources than when grown on DSB, and these results are in agreement with the present study [4]. The substrate acts as both an adhesion surface and as fermentable biomass for the fungi, and it activates the synthesis of hydrolytic complexes.

The increased FPase activity indicated the enhanced ability of the fungus to metabolize and degrade compounds in the biomass. The maximum activity was observed between 72 and 96 h, after which the activity was repressed due to the formation of degradation products.

To identify the origin of the enzymatic activity in the extracts, we evaluated the transcriptome expression at 96 h of fermentation.

### Sequencing assembly

In total, 246 million raw sequencing reads were generated with a target length of 72 bp (Table 1). After quality trimming, 84.11% of the data were retained for a total of 14.7 Gbp of sequencing data. *De novo* assembly using trimmed reads from all libraries resulted in 32,494 contigs, with an N50 of 1,251 bp. The assembled transcripts redundancy was determined through CD-HIT-EST. After this analysis, the final number of contigs was 32,396.

**Table 1:** Results of next-generation sequencing, trimming analysis, *de novo* assembly and mapping.

Library	DSB	CEL	LAC	Length (bp)	N50
**Number of Raw reads**	81,705,758	84,301,646	80,468,986	**72.0**	
**Number of trimmed reads**	**68,720,401**	**68,644,205**	**67,912,155**	**71.8**	
Single reads	5,820,501	6,451,047	5,586,409		
Paired reads	62,899,900	62,193,158	62,325,746		
**Number of Mapped reads**	**28,377,065**	**28,663,344**	**29,343,868**		
Single reads	2,463,163	2,803,982	2,477,029		
Paired reads	25,913,902	25,859,362	26,866,839		
**Unmapped reads**	**40,343,336**	**39,980,861**	**38,568,287**		
Single reads	3,357,338	3,647,065	3,109,380		
Paired reads	36,985,998	36,333,796	35,458,907		
**Contigs**	**32,494**				**1,251**
**CD-HIT-EST contigs**	**32,369**				

The raw reads were *de novo* assembled to generate contigs for further analysis and annotation.

### Analysis of the transcriptome under the influence of sugarcane bagasse as a substrate

**Figure 3:**
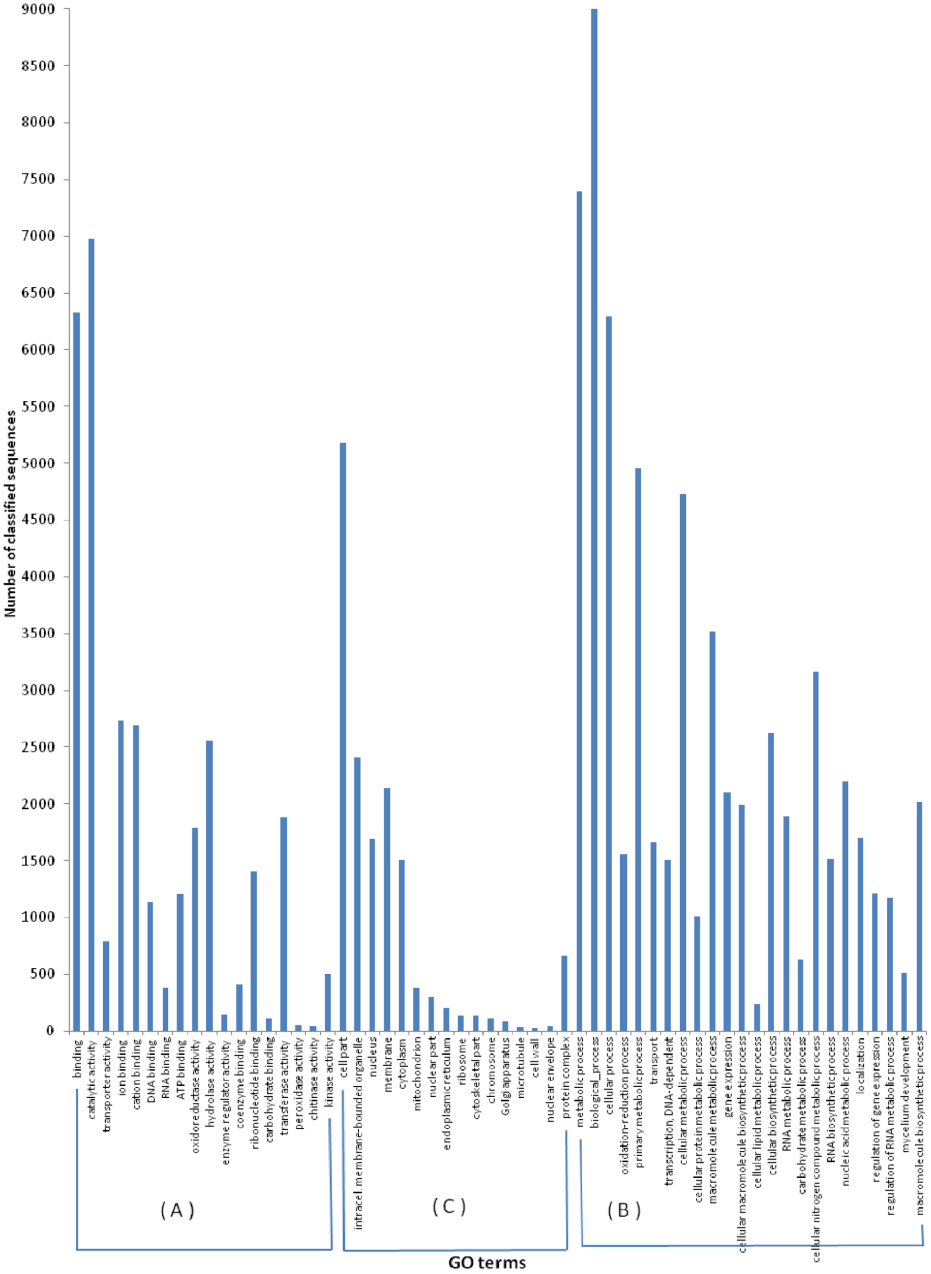
Molecular functions, biological process distribution and cellular localization of the transcriptome assembly. Contigs were assigned putative classifications based on homology and evaluated for their predicted involvement in molecular functions (A), biological processes (B) and cellular localization (C).

Transcript profiling is an important strategy for studying the expression of large gene sets under particular conditions. To determine the influence of the complex sugarcane bagasse substrate on gene expression, the contigs generated from the *de novo* assembly of the transcriptome were analyzed. The generated assembly was compared with the archive of the assembly scaffolds for *T. harzianum* CBS 226.95 [38], which demonstrated a similarity of 96% with the contigs derived from the assembly of the transcriptome. To identify the responses of the transcriptome under the different conditions tested, the contigs were annotated and classified according to their predicted functions (Figure 3).

The high number of sequences generated in this study, which were produced only through the use of next-generation sequencing, allows a precise overview of the different biological processes that occur in an organism at a given moment, and classifying these sequences allows for analysis of the genes that may be involved in biomass degradation. Sequences that were classified as possessing catalytic activity (6,975) or regulating enzymatic activity (143) may be involved in biomass degradation. When analyzed according to biological processes, the majority of the annotations were classified as participating in metabolic processes (7,393), followed by cellular processes (6,294). Regarding molecular functions, binding and catalytic activity were the most frequent classifications. Concerning cellular components, genes involved in cellular (5,184) and organelle (2,665) components and the membrane (2,143) were the most abundant.

**Figure 4:**
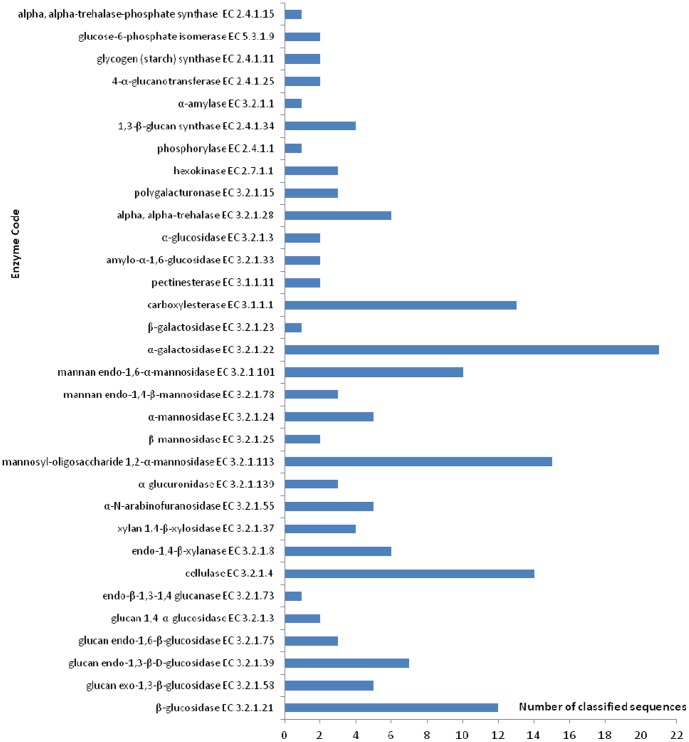
Identified sequences that catalyze reactions that are potentially involved in biomass degradation. The results of identification based on homology using the NCBI NR database indicate the presence of genes that are related to the depolymerization of biomass in the transcriptome.

For the hydrolysis of complex substrates such as sugarcane bagasse, a microorganism must produce an array of specialized enzymes that can hydrolyze lignocelluloses. The interaction between different classes of enzymes has been extensively studied [12],[13] and was observed in the present study. Figure 4 summarizes several of the enzyme classes that are potentially involved in biomass degradation and the number of contigs assigned to each of them. Of the contigs formed after assembly, 36.18% were classified according to GO terms, with 21.46% being involved in catalytic reactions. Approximately 164 contigs were classified as being potentially involved in metabolic reactions related to biomass degradation.

The β-glucosidase classification are specifically involved in the hydrolysis of cellulose. These sequences catalyze the hydrolysis of terminal, nonreducing β-D-glucose residues through the release of β-D-glucosidase (EC 3.2.1.21) and glucan 1,4-α-glucosidase (EC 3.2.1.3), which in turn catalyze the hydrolysis of terminal (1→4)-linked α-D-glucose residues from the nonreducing ends of the chains. Both of these steps release β-D-glucose, which is the monomer that is further metabolized.

Hemicellulose possesses a more varied composition than cellulose and requires enzymes to be effectively hydrolyzed. Sequences were classified as being involved in the degradation of xylan to monomers, including both endo-xylanases (EC 3.2.1.8), which cleave the xylanbackbone into shorter oligosaccharides, and β-xylosidase (EC 3.2.1.37), which cleaves short xylo-oligosaccharides into xylose. Similarly, sequences were related to mannan degradation (EC 3.2.1.113, EC 3.2.1.25, EC 3.2.1.24, EC 3.2.1.78 and EC 3.2.1.101). Several ancillary enzymes were also identified, including α-glucuronidase (EC 3.2.1.139), α-galactosidase (EC 3.2.1.22) and arabinofuranosidase (EC 3.2.1.55).

Systematic synergisms between the different enzyme classes could be observed for specific metabolic pathways in the *T. harzianum* transcriptome. These pathways included the metabolism of different sugars, which are associated with the depolymerization of biomass and were classified according to specific criteria of the Kyoto Encyclopedia of Genes and Genomes (KEGG) (Table 2) [28].

**Table 2:** Metabolic pathways expressed in different libraries.

Metabolic Pathway	Library	Enzyme Code Classification
Fructose and mannose metabolism	DSB	EC 1.1.1.17: 5-dehydrogenase
	CEL	EC 3.2.1.78: endo-1,4-β-mannosidase
Lysine biosynthesis	DSB	EC 1.2.1.31: dehydrogenase
Lysine degradation	DSB	EC 1.2.1.31: dehydrogenase
Purine metabolism	CEL	EC 2.7.7.7: DNA polymerase
	CEL	EC 3.6.1.3: adenylpyrophosphatase
	CEL	EC 3.6.1.15: nucleoside triphosphate phosphohydrolase
	LAC	EC 3.6.1.3: adenylpyrophosphatase
Thiamine metabolism	CEL	EC 3.6.1.15: nucleoside triphosphate phosphohydrolase
Methane metabolism	CEL	EC 1.14.13.8: monooxygenase
	CEL	EC 1.11.1.7: lactoperoxidase
Phenylpropanoid biosynthesis	CEL	EC 1.11.1.7: lactoperoxidase
Phenylalanine metabolism	CEL	EC 1.11.1.7: lactoperoxidase
Glycerophospholipid metabolism	CEL	EC 4..1.1.65: decarboxylase
	LAC	EC 1.1.1.8: dehydrogenase (NAD+)
Pyrimidine metabolism	CEL	EC 2.7.7.7: DNA polymerase
Starch and sucrose metabolism	CEL	EC 3.2.1.1: endo-1,4-β-D-glucanase
	LAC	EC 3.2.1.37: 1,4-β-xylosidase
Drug metabolism: cytochrome P450	CEL	EC 1.14.13.8: monooxygenase
Drug metabolism: other enzymes	LAC	EC 3.1.1.1: aliesterase
Riboflavin metabolism	CEL	EC 1.1.1.193: reductase
Fructose and mannose metabolism	CEL	EC 3.2.1.78: endo-1,4-β-mannosidase
Amino sugar and nucleotide sugar metabolism	LAC	EC 3.2.1.37: 1,4-β-xylosidase
		EC 3.2.1.55: arabinosidase
Pyruvate metabolism	LAC	EC 2.3.3.9: synthase
Nitrogen metabolism	LAC	EC 1.9.3.1: oxidase
Other glycan degradation	LAC	EC 3.2.1.45: psychosine hydrolase
Glycerolipid metabolism	LAC	EC 1.1.1.72: dehydrogenase (NADP+)
Oxidative phosphorylation	LAC	EC 1 1.9.3.1: oxidase
Glyoxylate and dicarboxylate metabolism	LAC	EC 2.3.3.9: synthase
Sphingolipid metabolism	LAC	EC 3.2.1.45:psychosine hydrolase
Fructose and mannose metabolism	DSB	EC 1.1.1.17: 5-dehydrogenase
	CEL	EC 3.2.1.78: endo-1,4-β-mannosidase
Lysine biosynthesis	DSB	EC 1.2.1.31: dehydrogenase
Lysine degradation	DSB	EC 1.2.1.31: dehydrogenase
Purine metabolism	CEL	EC 2.7.7.7: DNA polymerase
	CEL	EC 3.6.1.3: adenylpyrophosphatase
	CEL	EC 3.6.1.15: nucleoside triphosphate phosphohydrolase
	LAC	EC 3.6.1.3: adenylpyrophosphatase
Thiamine metabolism	CEL	EC 3.6.1.15: nucleoside triphosphate phosphohydrolase
Methane metabolism	CEL	EC 1.14.13.8: monooxygenase
	CEL	EC 1.11.1.7: lactoperoxidase
Phenylpropanoid biosynthesis	CEL	EC 1.11.1.7: lactoperoxidase
Phenylalanine metabolism	CEL	EC 1.11.1.7: lactoperoxidase
Glycerophospholipid metabolism	CEL	EC 4..1.1.65: decarboxylase
	LAC	EC 1.1.1.8: dehydrogenase (NAD+)
Pyrimidine metabolism	CEL	EC 2.7.7.7: DNA polymerase
Starch and sucrose metabolism	CEL	EC 3.2.1.1: endo-1,4-β-D-glucanase
	LAC	EC 3.2.1.37: 1,4-β-xylosidase
Drug metabolism: cytochrome P450	CEL	EC 1.14.13.8: monooxygenase
Drug metabolism: other enzymes	LAC	EC 3.1.1.1: aliesterase
Riboflavin metabolism	CEL	EC 1.1.1.193: reductase
Fructose and mannose metabolism	CEL	EC 3.2.1.78: endo-1,4-β-mannosidase
Amino sugar and nucleotide sugar metabolism	LAC	EC 3.2.1.37: 1,4-β-xylosidase
		EC 3.2.1.55: arabinosidase
Pyruvate metabolism	LAC	EC 2.3.3.9: synthase
Nitrogen metabolism	LAC	EC 1.9.3.1: oxidase
Other glycan degradation	LAC	EC 3.2.1.45: psychosine hydrolase
Glycerolipid metabolism	LAC	EC 1.1.1.72: dehydrogenase (NADP+)
Oxidative phosphorylation	LAC	EC 1 1.9.3.1: oxidase
Glyoxylate and dicarboxylate metabolism	LAC	EC 2.3.3.9: synthase
Sphingolipid metabolism	LAC	EC 3.2.1.45: psychosine hydrolase

The classification of the contigs according to the NCBI NR and KEGG databases [46],[28] indicates which metabolic pathway is active under a specific culture condition.

In this manner, different classes of enzymes that act together to degrade the cellulose backbone were identified. The application of enzymes to catalyze the degradation of cellulose to glucose and heteroxylans to pentose is now considered to be the most viable strategy for providing cost-efficient second-generation ethanol processes [39],[18], and the present study confirms that a variety of different metabolic pathways are necessary for sugar degradation in this yeast.

### Classification according to CAZymes

**Figure 5:**
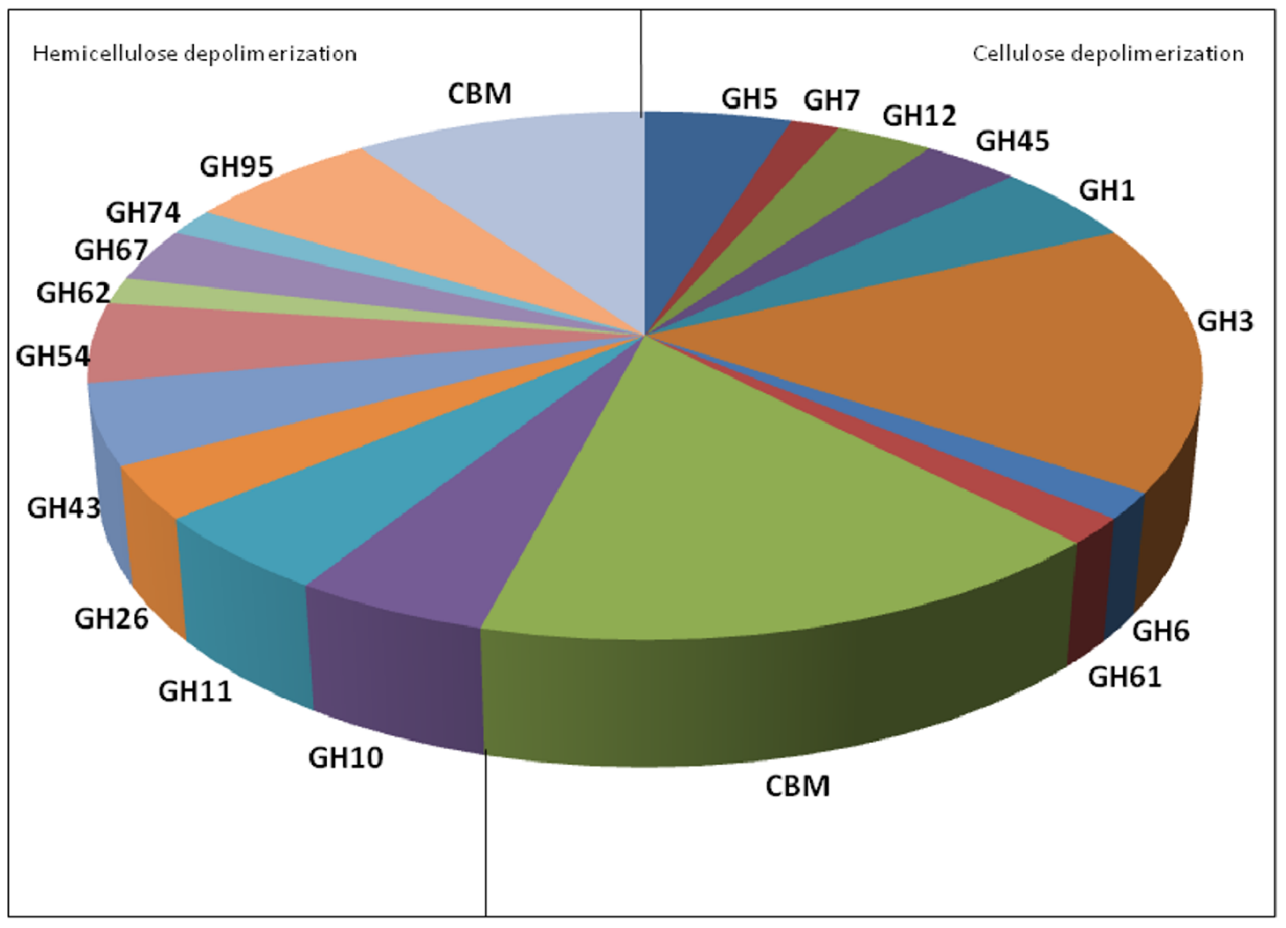
Encoded genes related to cellulose and hemicellulose depolymerization. Genes classified in the transcriptome analysis of *T. harzianum* fermentation on sugarcane bagasse.

To determine the number of encoded genes related to biomass degradation in the transcriptome, we searched for the following carbohydrate-active enzyme groups: glycoside hydrolases (GHs), glycosyltransferases (GTs), carbohydrate esterases (CEs) and the corresponding carbohydrate-binding modules (CBMs). We compared the transcriptome with a group of annotated sequences from the CAZymes database, including the annotated sequences for 17 cellulases from *T. reesei*. To identify CAZymes in *T. harzianum* IOC-3844, the assembled transcriptome was aligned against the specific CAZy dataset, and only the best alignment was considered for each gene sequence. A total of 527 CAZymes were identified in the *T. harzianum* IOC-3844 predicted gene set (unpublished results). We identified 487 predicted CAZymes in the transcriptome using a cutoff *E*-value of 1×10^−5^. From the predicted CAZymes, we identified 23 genes that encoded proteins of the glycoside hydrolase families that are involved in cellulose depolymerization: three genes were classified as GH5, one gene as GH7, two genes as GH12, two genes as GH45, three genes as GH1, 10 genes as GH3, one gene as GH6 and one gene as GH61. In the cellulose depolymerization group, we found 10 different sequences that encoded carbohydrate-binding modules. Regarding genes involved in hemicellulose depolymerization, 22 genes were identified from the following glycoside hydrolase families: three genes from GH10, three genes from GH11, two genes from GH26, three genes from GH43, three genes from GH54, one gene from GH62, two genes from GH67, one gene from GH74 and four genes from GH95. Six carbohydrate-binding module sequences were classified as belonging to enzymes that degrade hemicellulose (Figure 5).

### Comparative expression analysis

To analyze the differences in expression levels among the tested growth conditions, we compared the total assembly generated from all of the sequenced transcriptome libraries (DSB+CEL+LAC library) with each individual transcriptome assembly. The mapping results are shown in Table 1.

**Figure 6:**
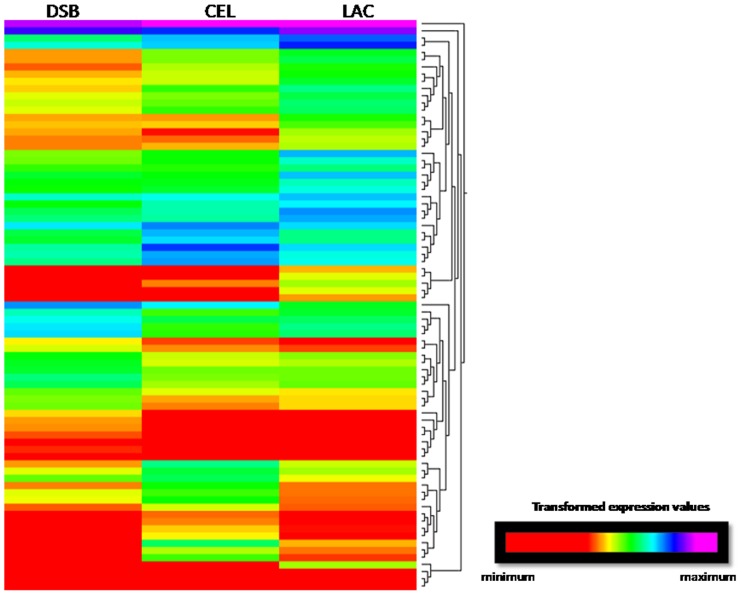
K-means clustering of differentially expressed genes identified as CAZymes (summarized in Table 3).

**Figure 7:**
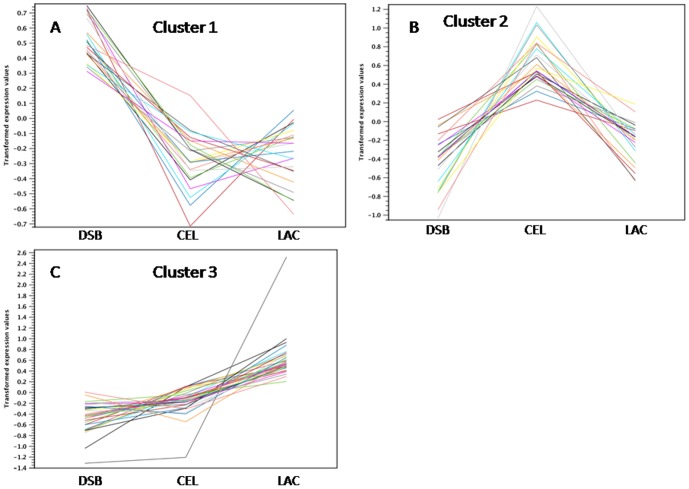
The identified genes (summarized in Table 3) were resolved into three clusters. The identified genes were further classified into one of three response profiles based on where they were most highly expressed: (A) in the DSB library, (B) in the CEL library or (C) in the LAC library.

To identify the transcriptomic responses under each condition, we analyzed the distribution of the genes that were identified as being differentially expressed. Pairwise comparisons of the subsets indicated the total number of genes that were overexpressed under each condition. The classification of differentially expressed contigs allowed us to determine the set of genes for carbohydrate-active enzymes that were upregulated in each group (Table 3, Figure 6 and 7).

**Table 3:** Classification of sequences present in upregulated groups, according to the CAZyme database.

Upregulated group	Cluster	Contig	Length (bp)	Lowest E-value	CAZy	RPKM		
						DSB	CEL	LAC
**DSB**	**1**	6765	1,521	0.00	GT69	58.25	28.90	29.42
		7294	1,155	3.72E-33	GT39	84.76	51.53	42.58
		14029	3,726	0.00	GT2	97.16	35.91	58.69
		15954	665	3.74E-13	GH28	64.39	28.07	32.29
		16314	1,044	0.00	GH76	144.84	88.02	98.48
		19203	218	1.86E-34	GH28	293.39	163.56	212.91
		19252	218	1.37E-47	GT2	81.63	42.09	33.30
		19677	250	1.52E-24	GT2	58.78	31.54	24.40
		20009	209	5.02E-35	GH16	103.86	68.61	58.86
		21947	1,631	0.00	GT2	194.11	123.70	142.81
		22621	1,184	0.00	GH125	151.61	80.77	97.54
		23959	898	0.00	GT20	251.35	144.52	151.95
		24020	330	2.94E-173	GT4	359.77	167.88	260.13
		24053	1,141	0.00	GT48	210.54	152.82	151.10
		24197	823	1.37E-36	CE11	156.59	115.13	101.66
		24491	696	0.00	GH128	332.95	226.17	240.71
		25186	416	2.98E-6	CE11	228.13	132.09	154.75
		27900	309	5.9E-42	GH78	373.50	176.02	247.26
		28105	267	2.72E-97	GH72	201.54	119.69	131.08
		28257	262	1.35E-19	GH1	70.75	37.95	31.09
		29726	1,876	0.00	GH5	118.25	83.09	68.79
		31930	361	3.11E-150	GH43	447.09	355.16	205.51
**CEL**	**2**	15484	1,124	0.00	GT24	37.62	129.12	77.71
		15510	596	0.00	GH5/CBM1	49.83	239.69	88.81
		15808	642	1.32E-64	GH5	54.56	95.81	60.99
		17441	1,93	5.04E-34	GH10	83.44	257.14	120.21
		19410	217	2.19E-39	GH3	24.20	49.84	17.90
		19509	227	3.56E-37	GT2	287.20	425.57	320.97
		19636	212	5.34E-29	GT2	38.56	103.35	54.65
		19662	220	7.85E-39	GT2	115.65	220.27	135.39
		20286	203	2.77E-26	GH11	954.08	2,397.6	1,835.5
		20620	1,993	9.99E-31	CE15	16.83	58.90	22.72
		23663	557	0.00	CBM20	257.18	433.50	330.20
		23934	1,045	0.00	GT2	221.66	416.88	256.88
		24061	1,256	0.00	GT2	115.31	164.11	77.22
		24114	526	0.00	GT48	363.92	467.54	372.60
		24258	1,118	0.00	GT2	111.80	187.01	75.47
		24717	1,283	0.00	GT2	279.09	550.73	376.59
		25735	222	5.84E-52	GT2	211.60	375.59	260.66
		26247	262	2.93E-56	GH18	153.47	228.37	111.86
		28387	203	2.13E-33	GH18	71.69	119.10	73.19
		31105	817	1.17E-141	GH10	47.92	166.37	67.24
		32239	223	3.49E-37	GT48	36.66	74.47	42.48
		5331	2,885	0.00	GH2/GH27	34.36	80.30	42.64
		6707	1,307	0.00	GH10	80.29	185.68	77.10
**LAC**	**3**	7215	1,907	0.00	GH43	78.81	91.29	130.49
		10859	1,914	0.00	GH47	193.40	198.30	294.08
		18143	324	2.36E-87	GH76	79.40	74.62	126.43
		18902	221	3.46E-37	CE5/CBM1	84.67	146.97	206.94
		19230	225	7.7E-45	GH18/CBM1	90.84	121.41	184.33
		19615	210	9.28E-38	CE5/CBM1	101.02	123.10	210.96
		20453	1,983	0.00	GH5	9.49	10.24	135.54
		22045	1,22	0.00	GH18	13.66	30.17	53.55
		22732	1,036	0.00	GH43/CBM1	73.44	129.52	179.90
		23357	1,136	0.00	CE5	32.57	49.05	88.74
		23867	1,884	0.00	GH55	187.33	207.94	318.03
		24034	1,109	0.00	GH2	192.75	286.72	354.68
		24118	1,48	0.00	GH3	122.62	153.22	248.20
		24121	392	5.3E-90	GH72/CBM43	242.90	397.74	516.40
		24529	582	2.79E-147	GH31	171.29	177.98	249.27
		24859	367	3.48E-109	GH62	148.35	184.33	413.59
		25106	622	0.00	CE5/CBM1	228.89	287.24	445.60
		25478	230	6.87E-92	GH3	87.18	84.79	179.73
		25634	386	1.23E-143	GH18	209.61	190.08	393.49
		26359	317	7.11E-105	GH18/CBM1	115.17	144.83	221.46
		26916	303	1.61E-158	GH31	113.16	169.49	238.66
		27290	226	2.98E-32	GH11	86.07	60.98	133.60
		27342	313	2.27E-116	GH62	35.46	47.15	115.85
		27456	265	4.58E-54	GH71/CBM24	49.47	79.91	132.71
		28080	593	2.34E-32	GH6	702.66	565.85	859.84
		28132	364	0.00	GH31	315.80	392.58	588.33
		28344	358	2.73E-110	GH11	83.67	144.72	220.94
		28732	330	5.55E-31	GH92	93.23	96.63	159.65
		29910	1,762	0.00	GH54/CBM42	48.08	58.71	115.27
		29947	1,601	0.00	CE5/CBM1	96.47	169.32	259.20
		30067	1,876	0.00	GH64	47.94	54.27	84.11
		31154	1	0.00	GH13/CBM48	307.18	336.14	398.07
		31183	505	0.00	GH62/CBM1	253.87	274.20	429.39

The sequences presenting expectation values lower than 1×10^−3^, and the best alignment scores are summarized. The clusters are classified according to Figure 6 and 7.

Different genes corresponded to different glycoside hydrolase families involved in carbohydrate metabolism in the different upregulated groups. According to the Carbohydrate-Active Enzymes database [40], the glycoside hydrolases of family 1 include enzymes that possess β-glucosidase (EC 3.2.1.21), β-galactosidase (EC 3.2.1.23) and β-mannosidase (EC 3.2.1.25) activities; the glycoside hydrolases of family 18 possess chitinase (EC 3.2.1.14) activity; the glycoside hydrolases of family 55 exhibit exo-b-1,3-glucanase (EC 3.2.1.58) and endo-b-1,3-glucanase (EC 3.2.1.39) activities; the glycoside hydrolases of family 3 exhibit β-glucosidase (EC 3.2.1.21) and xylan 1,4-β-xylosidase (EC 3.2.1.37) activities; the glycoside hydrolases of family 5 possess chitosanase (EC 3.2.1.132), b-mannosidase (EC 3.2.1.25), endo-b-1,4-glucanase/cellulase (EC 3.2.1.4) and glucan b-1,3-glucosidase (EC 3.2.1.58) activities; the glycoside hydrolases of family 11 present endo-1,4-β-xylanase (EC 3.2.1.8) activity; and the glycoside hydrolases of family 16 exhibit endo-1,3-β-glucanase (EC 3.2.1.39) or endo-1,3(4)- β-glucanase (EC 3.2.1.6) activity. The LAC library contained 33 classified genes, whereas the CEL library contained 23 genes and the DSB library contained 22 genes. These gene classifications included glycosyltransferases (GTs), which catalyze the transfer of sugar moieties from activated donor molecules to specific acceptor molecules to form glycosidic bonds; carbohydrate esterases (CEs); and the corresponding carbohydrate-binding modules (CBMs). Glycosyltransferases can be classified as either retaining or inverting enzymes according to the stereochemistry of their substrates and reaction products. The glycosyltransferases of family 2 (GT2) exhibit cellulose synthase (EC 2.4.1.12) and chitin synthase (EC 2.4.1.16) activities and appear in all three libraries. The glycosyltransferases of family 4 (GT4) exhibit sucrose synthase (EC 2.4.1.13) and sucrose-phosphate synthase (EC 2.4.1.14) activities. Therefore, some of the genes that are responsible for biomass degradation reactions are highly expressed, whereas others, though not highly expressed, may also confer the ability to degrade organic compounds for energy in this fungus. Thus, the fungus can adapt its cellulolytic system to the composition of its medium by increasing or decreasing the expression of certain genes, as observed in the present study.

## Discussion

The ability of filamentous fungi to efficiently degrade plant polymers is an important aspect of microbial ecology and may afford many potential industrial applications. The fungal strain *T. harzianum* demonstrates promising results for on-site cellulase production and is a potential candidate for the production of hydrolytic enzymes [4],[6].

To evaluate the cellulase activity of this fungus on pretreated sugarcane bagasse, we measured FPase, which reflects the overall activity of multicomponent enzyme complexes for cellulose hydrolysis [41]. An increase in cellulose activity is observed over the course of cultivation until 96 h, which represents the maximum cellulolytic activity (Figure 2). The DSB sample, which was used as the inducer, initiated fermentation at a level 4-fold greater than cellulolytic activity, which is most likely due to previous adaptation of the fungus to the substrate during the production of mycelia (during the preculture). In this case, the set of genes that were activated during the induction of mycelial growth was identical to the set used in fermentation, which allowed for a higher rate of fermentation in the first 24 h. In the first 48 to 96 h, the cellulolytic activity profile of the sample induced with DSB maintained a growth profile and FPase that was statistically similar to that of samples induced with cellulose. This result indicates that the set of genes that were active after the adaptation phase of fermentation may have been similar between the samples; however, the sample induced with DSB must have differentially expressed some genes in the first 24 h of growth because it reached a higher peak of cellulolytic activity (0.2±0.01 FPU mL^−1^) compared with the samples induced with cellulose and lactose. The sample that used lactose as the inducer of mycelial growth maintained lower levels of activity throughout the fermentation. Notably, in the first 24 h of fermentation, the CEL and LAC samples both achieved similar (0.05±0.004 and 0.02±0.002 FPU mL^−1^, respectively) levels of FPase, suggesting that the set of genes that were activated during the preculture phase generated similar rates of cellulose-degrading enzymatic activity.

To elucidate how the complex sugarcane bagasse substrate influences the set of fungal gene transcripts that conferred enzymatic activity, we analyzed the transcription profiles of the samples. The results represent the first characterization of global gene expression in *T. harzianum* grown on a complex substrate (Figure 3). In the analysis of 32,494 contigs from the cDNA library, 6,975 sequences were classified as possessing catalytic activity (21.46% of total contigs), of which 2,555 possess hydrolase activity and act on chemical bonds such as ester, carbon-nitrogen and carbon-carbon bonds (Table 4). The high number of identified hydrolase sequences allowed us to determine the gene sequences that were related to specific degradation reactions. A similar annotation profile, which was generated using Gene Ontology (GO), was described by Steindorff *et al.* (2012) [5] for an EST sequencing library of 2,927 high-quality sequences. In both experiments, catalytic activity and binding represented the major classified molecular functions, with metabolic and cellular processes being the most prevalent classifications, and the cell and organelle category constituting the most common cellular localization.

**Table 4:** Contig sequences classified according to their putative hydrolytic activity.

GO ID	TERM	Number of classified sequences
GO:0016787	hydrolase activity	2,555
GO:0004553	hydrolase activity, hydrolyzing O-glycosyl compounds	336
GO:0016788	hydrolase activity, acting on ester bonds	494
GO:0016798	hydrolase activity, acting on glycosyl bonds	373
GO:0016818	hydrolase activity, acting on acid anhydrides, in phosphorus-containing anhydrides	778
GO:0016798	hydrolase activity, acting on glycosyl bonds	373
GO:0016818	hydrolase activity, acting on acid anhydrides, in phosphorus-containing anhydrides	778
GO:0016817	hydrolase activity, acting on acid anhydrides	789
GO:0016810	hydrolase activity, acting on carbon-nitrogen (but not peptide) bonds	140
GO:0016820	hydrolase activity, acting on acid anhydrides, catalyzing transmembrane movement of substances	174
GO:0042578	phosphoric ester hydrolase activity	155
GO:0052689	carboxylic ester hydrolase activity	77
GO:0017171	serine hydrolase activity	113
GO:0008081	phosphoric diester hydrolase activity	54
GO:0016811	hydrolase activity, acting on carbon-nitrogen (but not peptide) bonds in linear amides	40
GO:0016790	thiolester hydrolase activity	45
GO:0008484	sulfuric ester hydrolase activity	17
GO:0016813	hydrolase activity, acting on carbon-nitrogen (but not peptide) bonds in linear amidines	17
GO:0016814	hydrolase activity, acting on carbon-nitrogen (but not peptide) bonds in cyclic amidines	25
GO:0047617	acyl-CoA hydrolase activity	9
GO:0033961	cis-stilbene-oxide hydrolase activity	8
GO:0016803	ether hydrolase activity	10
GO:0016289	CoA hydrolase activity	9
GO:0016801	hydrolase activity, acting on ether bonds	11
GO:0019238	cyclohydrolase activity	9
GO:0004416	hydroxyacylglutathione hydrolase activity	4
GO:0016799	hydrolase activity, hydrolyzing N-glycosyl compounds	5
GO:0019120	hydrolase activity, acting on acid halide bonds, in C-halide compounds	3
GO:0003933	GTP cyclohydrolase activity	5
GO:0004848	ureidoglycolate hydrolase activity	3
GO:0003935	GTP cyclohydrolase II activity	3
GO:0004477	methenyltetrahydrofolate cyclohydrolase activity	2
GO:0004045	aminoacyl-tRNA hydrolase activity	2
GO:0003934	GTP cyclohydrolase I activity	2
GO:0008474	palmitoyl-(protein) hydrolase activity	2
GO:0004463	leukotriene-A4 hydrolase activity	2
GO:0016823	hydrolase activity, acting on acid carbon-carbon bonds in ketonic substances	3
GO:0016824	hydrolase activity, acting on acid halide bonds	3
GO:0016822	hydrolase activity, acting on acid carbon-carbon bonds	3
GO:0004039	allophanate hydrolase activity	1
GO:0004635	phosphoribosyl-AMP cyclohydrolase activity	1
GO:0004649	poly(ADP-ribose) glycohydrolase activity	1
GO:0033971	hydroxyisourate hydrolase activity	1
GO:0018738	S-formylglutathione hydrolase activity	1
GO:0004301	epoxide hydrolase activity	1
GO:0033699	DNA 5′-adenosine monophosphate hydrolase activity	1
GO:0003937	IMP cyclohydrolase activity	1
GO:0016802	trialkylsulfonium hydrolase activity	1
GO:0016815	hydrolase activity, acting on carbon-nitrogen (but not peptide) bonds in nitriles	1

The current study identified genes that were upregulated by different substrates in the preculture phase (Table 3). The DSB library contained 792 classified contigs, 514 of which were homologous to the *T. harzianum* genome and 22 of which were related to the CAZyme library. Among the 377 classified contigs in the CEL library, 243 were related to the genome, and 23 were identified among the CAZyme. Among the 299 classified contigs in the LAC library, 272 genes were related to the genome, and 33 were identified in the CAZyme dataset (Table 3). Therefore, according to the CAZyme classification, 79 genes were differentially expressed between two conditions and exhibited an expression level that was measurable in the other conditions. In this analysis, the gene expression values fell into three profiles after K-means clustering (Figure 6). Cluster 1 (Figure 7A) contained the genes (members of the glycoside hydrolase family) that were most highly expressed in the DSB library; cluster 2 (Figure 7B) contained the most highly expressed genes in the CEL library and.Cluster 3 (Figure 7C) contained the most highly expressed genes in the LAC library; A difference observed between the set group of GHs could be related to the influence of the different substrates. Several contigs were analyzed further in terms of their expression values and similarity.

Among the differentially expressed genes, we identified genes related to extracellular degradative enzymes that play an important role in pathogenesis. These enzymes include the carbohydrate esterase family 5 protein, whose cutinase domain (contig 25106, classified based on CAZ similarity as EHK47149.1, IPR000675) hydrolyzes cutin and facilitates fungal penetration through the cuticle. Inhibition of this enzyme can prevent fungal infection through intact cuticles. When cutin monomers are released from the cuticle due to small amounts of cutinase on fungal spore surfaces, these monomers can greatly increase the amount of cutinase secreted by the spore, although the mechanism underlying this process remains unknown. Another, more highly expressed, contig was classified as a member of the GH11 family (contig 20286).The overwhelming majority of the glycoside hydrolases of this family are xylanases. These enzymes carry out the endohydrolysis of (1→4)-beta-D-xylosidic linkages in xylans and random hydrolysis of (1→3)-beta-D-glycosidic linkages in (1→3)-beta-D-xylans. Contig 27456 exhibited similarity to family GH71, which includes α-1,3-glucanase (EC 3.2.1.59). *O*-Glycosyl hydrolases (EC 3.2.1.) are a widespread group of enzymes that hydrolyze glycosidic bonds between two or more carbohydrates or between a carbohydrate and a noncarbohydrate moiety (IPR005197), and they are also related to CBM24 (α-1,3-glucan (mutant)-binding function) [42].

In the DSB library, contig 20009 was found to be differentially expressed at a significant RPKM level (103.86) and was similar to a GH16 protein from *Trichoderma virens* (EHK18881.1, IPR000757). The GH16 family contains a variety of enzymes with a range of known activities. Lichenase (EC 3.2.1.73), xyloglucan xyloglucosyltransferase (EC 2.4.1.207), agarase (EC 3.2.1.81), kappa-carrageenase (EC 3.2.1.83), endo-β-1,3-glucanase (EC 3.2.1.39), endo-β-1,3-1,4-glucanase (EC 3.2.1.6) and endo-β-galactosidase (EC 3.2.1.103) are all members of this family.

In this study, we identified sequences related to different classes of enzymes that act on the cellulose backbone, such as GH5, which exhibits endo-β-1,4-glucanase activity in *T. reesei* (EC 3.2.1.4) and is responsible for the hydrolysis of the (1→4)-β-D-glucosidic linkages in cellulose. The GH3 family exhibits β-glucosidase activity in *T. reesei* (EC 3.2.1.21), where it hydrolyzes terminal, nonreducing β-D-glucosyl residues and releases β-D-glucose. This monomer can enter into the eukaryotic energy pathway of glycolysis. Glycolysis produces energy and requires an input of two ATP molecules. This input is used to generate four new ATP molecules, resulting in a net gain of two ATP molecules. Two NADH molecules are also produced; these molecules serve as electron carriers for other biochemical reactions in the cell. The enzymes that are necessary to catalyze the degradation of glucose molecules are expressed throughout the growth of the fungus on the complex substrate, possibly to produce energy through glycolysis and support cell survival and reproduction. The enzymes that act in biomass degradation were the focus of this work, and this analysis allowed us to identify a set of enzymes that are involved in carbohydrate metabolism based on expression profiles.

Regarding expression differences, the LAC library contained numerous genes receiving CAZyme classifications. Lactose, an inexpensive, soluble substrate, leads to reasonably good induction for cellulase production [43],[44]. The fungus does not directly take up lactose but instead hydrolyzes the compound to galactose and glucose. Cellulase synthesis cannot be induced by galactose, and the addition of galactose to the medium decreases FPase levels in the supernatant [45], as reported in this work. Karaffa *et al.* [43] reported that lactose induces significantly higher cellulase levels compared to galactose, but galactose induces cellulase gene expression at low growth rates in *T. reesei*. In this study, the highest degradation rate would have occurred in the fungi precultured on the lactose medium, followed by cellulose and DSB, due to the complexity of the substrate and the stability of the organic chains involved. The presence of lactose in the early stages of the experiment would have induced genes that are sensitive to lactose and galactose, which may explain the low level of FPase observed in the sample that used lactose as the inducer of hydrolytic systems (even in the fermentation step). As shown in the results, sugarcane bagasse was able to activate the expression of a different set of genes that were differentially expressed compared with the control, and this difference was associated with an increase in cellulose enzymatic activity during fermentation.

This strain of *T. harzianum* demonstrates a complex and efficient genetic mechanism for biomass degradation. The use of RNA-Seq technology was shown to be an efficient strategy for the discovery and selection of potential target genes. The results reported here are valuable for further studies on the expression, purification and characterization of recombinant enzymes for efficient cellulose degradation.

## Supporting Information

Table S1 Primers used for RT-qPCR detection of glycosyl hydrolase genes. The squalene-epoxidase gene was used as endogenous control and the sequences analyzed encoded genes of glycoside hydrolases (GHs), carbohydrate esterases (CEs) and carbohydrate-binding modules (CBM).
(XLSX)
